# Predicting pH-Dependent Solubility Enhancement and Precipitation Suppression in Drug–Cyclodextrin–Arginine Formulations

**DOI:** 10.3390/pharmaceutics18070834

**Published:** 2026-07-07

**Authors:** Natalia Bolocan, Igor Povar, Alina Catrinel Ion, Oxana Spinu

**Affiliations:** 1 Institute of Chemistry, Moldova State University, MD-2028 Chisinau, Moldova; natalia.bolocan@ancd.gov.md (N.B.); oxana.spinu@sti.usm.md (O.S.); 2 Faculty of Applied Chemistry and Materials Science, University POLITEHNICA of Bucharest, 011061 Bucharest, Romania

**Keywords:** cyclodextrins, ternary systems, L-arginine, solubility enhancement, precipitation suppression, pH-dependent solubility, inclusion complexation, supersaturation, formulation development

## Abstract

**Background/Objectives**: Cyclodextrin-based ternary systems are widely used to improve the solubility of poorly soluble drugs. Amino acids such as L-arginine may further increase dissolved drug concentrations and reduce precipitation under physiologically relevant conditions. In many systems, apparent solubility enhancement is influenced simultaneously by drug ionization, inclusion complex formation, multicomponent interactions, and solid–liquid equilibria. This study presents a physicochemical modeling approach for analyzing pH-dependent solubility enhancement and precipitation behavior in drug–cyclodextrin–L-arginine systems. **Methods**: The model combines acid–base equilibria, binary inclusion complexation, ternary association, and explicit solid-phase partitioning within a unified mass-balance treatment. The approach was applied to representative ternary systems containing repaglinide, sulfadiazine, cefixime, and meloxicam. **Results**: Quantitative comparison with published phase-solubility data for the repaglinide–HPβCD–L-arginine system confirmed the numerical consistency of the model. The calculated profiles showed that enhanced solubilization and reduced precipitation occur only within specific pH regions determined by coupled equilibrium effects. For cefixime and meloxicam, the calculations were interpreted as predictive applications because directly comparable validation datasets were not available. Outside the favorable pH regions, a substantial fraction of the drug remained in the solid phase. **Conclusions**: These observations support the importance of pH and multicomponent interactions in controlling formulation performance in cyclodextrin-containing systems. The obtained profiles may support preliminary optimization of formulation pH and excipient composition before experimental screening.

## 1. Introduction

Poor aqueous solubility remains one of the major limitations in the development of formulations for Biopharmaceutics Classification System (BCS) Class II drugs, where dissolution frequently controls oral absorption and bioavailability [1,2]. Cyclodextrins (CDs) are widely used pharmaceutical excipients that improve the apparent solubility and dissolution behavior of poorly soluble drugs through inclusion complex formation with hydrophobic regions of drug molecules while maintaining compatibility with aqueous media [3,4,5]. Their pharmaceutical use includes solubilization, stabilization, enhancement of drug delivery, and modification of release properties [3,4,5]. Phase-solubility analysis introduced by Higuchi and Connors remains the classical approach for evaluating drug–cyclodextrin interactions under saturated conditions [6], while complementary solid-state techniques are commonly used to characterize inclusion complexes [7].

Additional improvement of drug solubility is frequently achieved by introducing auxiliary agents such as amino acids, hydroxy acids, or hydrophilic polymers into cyclodextrin-based systems [4,5]. Among these additives, L-arginine has attracted considerable attention because it may simultaneously increase dissolved drug concentrations and reduce precipitation tendency in aqueous media [8,9,10,11]. L-arginine was selected because it is a pharmaceutically relevant basic amino acid that has been used as an auxiliary component in cyclodextrin-based solubilization studies [8,9,10,11]. Its guanidinium group remains protonated over a broad pH range [12,13] and may participate in electrostatic interactions, ion-pair formation, and hydrogen bonding with ionized acidic drugs and cyclodextrin-associated species [8,9,10,11,14]. In addition, the available literature provides equilibrium and phase-solubility data for L-arginine-containing systems, which makes it suitable for quantitative modeling. Other amino acids or excipients may also enhance cyclodextrin complexation, but their use in the present framework would require their own association constants under comparable conditions. Experimental studies involving repaglinide, sulfadiazine, cefixime, and meloxicam have shown that ternary systems containing cyclodextrins and L-arginine may provide substantially higher apparent solubility than the corresponding binary drug–cyclodextrin systems [8,9,10,11]. These effects are commonly associated with improved complexation efficiency and stabilization of dissolved drug species.

Increased apparent solubility does not necessarily guarantee maintenance of stable dissolved drug concentrations after dilution or changes in pH. Under gastrointestinal conditions, precipitation may occur when the formulation enters unfavorable solubilization regions. Formulation performance therefore depends on equilibrium solubility together with ionization equilibria, complex formation, and solid–liquid phase distribution. For ionizable drugs, pH strongly affects the relative abundance of neutral and charged species and directly influences both binary inclusion and multicomponent association processes.

Ternary cyclodextrin systems have been extensively investigated experimentally, and interpretation of their behavior remains difficult because several coupled processes may contribute simultaneously to the observed enhancement of apparent solubility. Increased dissolved drug concentrations may originate from pH-induced ionization, binary inclusion complexation, multicomponent association, or combinations of these effects. Optimization of ternary formulations therefore still relies largely on empirical screening.

Recent studies have emphasized the importance of pH-dependent solubility, supersaturation behavior, precipitation processes, and multicomponent stabilization effects in cyclodextrin-containing pharmaceutical formulations [15,16,17]. These studies illustrate the growing interest in predictive approaches capable of relating coupled physicochemical interactions to formulation performance under complex conditions. Recent thermodynamic analyses of heterogeneous multicomponent systems also showed that apparent synergistic enhancement may arise from coupled equilibria involving simultaneous protonation, association, and phase partitioning processes [18,19,20]. Such observations indicate the importance of distinguishing pH-driven enhancement from stabilization associated with multicomponent interactions during interpretation of complex formulation systems.

The developed model combines acid–base equilibria, binary inclusion complexation, ternary association, and explicit solid–liquid phase partitioning within a unified mass-balance treatment. In addition to apparent solubility, the analysis introduces formulation-relevant descriptors related to precipitation degree and multicomponent enhancement. The approach is evaluated using literature experimental data for repaglinide, sulfadiazine, cefixime, and meloxicam, representing structurally distinct poorly soluble drugs known to form ternary cyclodextrin systems. The objective of the study is to provide a predictive equilibrium-based approach capable of identifying formulation-relevant pH domains associated with enhanced solubility and reduced precipitation. The present study applies equilibrium analysis to predict pH-dependent solubility enhancement and precipitation behavior in drug–cyclodextrin–L-arginine systems. The proposed approach may assist preliminary identification of formulation-relevant pH regions and excipient compositions prior to experimental optimization.

## 2. Materials and Methods

### 2.1. Model Systems and Physicochemical Equilibria

The analyzed systems consisted of a poorly soluble ionizable drug (HD), cyclodextrin (CD), and L-arginine (HA) used as an auxiliary solubilizing component. Under saturated conditions, the total drug content was distributed between dissolved species and a residual solid phase. Drug ionization was described through conventional acid–base equilibria accounting for the coexistence of neutral and ionized forms as a function of pH. L-arginine was treated as a polyprotic component whose protonation state changes with pH and influences participation in ternary interactions.

Binary drug–cyclodextrin complex formation was represented as a reversible equilibrium process. Additional solubility enhancement in ternary systems was described through drug–cyclodextrin–arginine association equilibria involving protonated arginine species. These coupled equilibria determine the availability of dissolved species participating in multicomponent stabilization.

The complete mathematical expressions describing acid–base equilibria, complex formation, and mass-balance equations are provided in Appendix A (Appendix A.1, Appendix A.2 and Appendix A.3).

The coupled equilibrium equations were solved numerically to obtain pH-dependent species distributions, apparent solubility profiles, and precipitation behavior under specified formulation conditions. The equilibrium constants used in the calculations were taken from experimental literature sources summarized in Table 1. The calculations used the equilibrium constants as reported in the original literature sources. No additional ionic-strength correction was applied because the necessary activity-coefficient parameters were not available consistently for all systems. Therefore, the predictions should be interpreted for conditions close to those under which the constants were determined.

For the RPG–HPβCD–L-arginine system, the source study used HPβCD with an average molecular weight of approximately 1377 and a hydroxypropyl substitution degree of 0.66. The calculated profiles for this system refer to this HPβCD material and to the experimental conditions reported in that study. Direct transfer of these calculations to HPβCD products with different substitution degrees would require the corresponding association constants.

The stereochemical form of each drug was taken as reported in the experimental source from which the equilibrium constants were obtained. Enantiomer-specific constants were not introduced. Predictions for other stereoisomers or racemic compositions would require independently measured constants under comparable conditions.

Because the investigated systems were analyzed under saturation conditions, the formulation explicitly included both dissolved species and the undissolved drug phase. Apparent solubility was therefore expressed as the total dissolved drug concentration at equilibrium, including free drug, ionized species, binary drug–cyclodextrin complexes, and ternary associations.

### 2.2. Solubility and Precipitation Calculations

The total concentration of each component was expressed through mass-balance equations including all relevant dissolved species and, for the drug, the residual solid phase. Apparent solubility $S_{app}$ was calculated as the sum of all dissolved drug forms at equilibrium:

$$S_{app}=\left[free\;drug\right]+\left[ionized\;species\right]+\left[binary\;complexes\right]+[ternary\;complexes]$$

Cyclodextrin was assumed to remain fully dissolved under the investigated conditions, and its analytical concentration was distributed between free and complexed forms. The CD–H_2_A^+^ association term was included in the cyclodextrin and L-arginine mass balances, as the corresponding association constant was available. A separate CD–HA term was not used in the calculations, since an independent constant measured under comparable conditions was not available for the analyzed systems. This interaction may affect complexation at moderate pH and can be added to the model once suitable constants are reported.

Similarly, the total concentration of L-arginine included all protonation states and ternary-associated species.

This treatment accounts simultaneously for the effects of pH, ionization, complex formation, and solid–liquid partitioning on formulation behavior. Consequently, changes in pH or component concentrations influence species distribution, complex stability, and the fraction of drug remaining in the solid phase.

The complete set of algebraic expressions used in the calculations is provided in Appendix A (Appendix A.2, Appendix A.3 and Appendix A.4). Additional thermodynamic interpretation and examples of multicomponent solubilization behavior are discussed in Appendix A (Appendix A.7).

**Figure 1:**
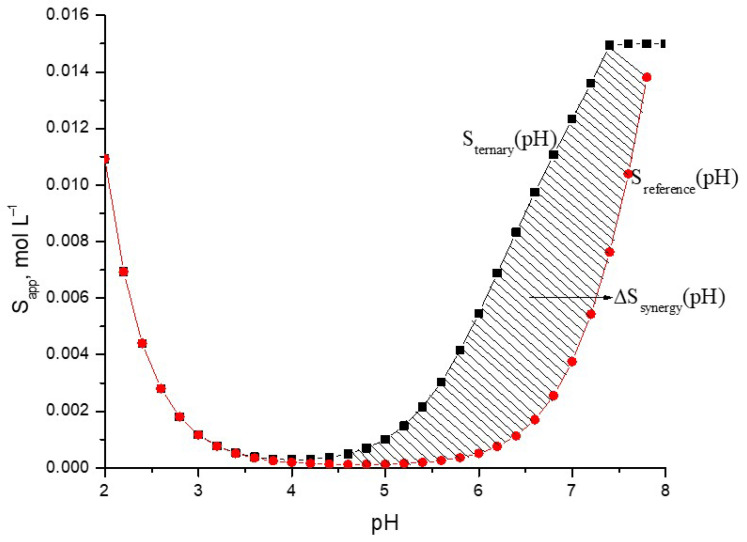
Deconvolution of apparent solubility in the ternary RPG–HP-β-cyclodextrin–L-arginine system into pH-driven and multicomponent contributions. The apparent solubility of the ternary system (black line) is compared with a reference system in which ternary association is suppressed (red line). The difference between the curves corresponds to the absolute solubility enhancement ($∆S_{synergy}(pH)$), identifying the pH region where multicomponent stabilization is most pronounced. Calculations were performed at equimolar concentrations of drug, cyclodextrin, and L-arginine (0.015 mol L^−1^).

The dependence of apparent solubility on pH and formulation composition was obtained from the coupled equilibrium calculations. Figure 1 illustrates the calculated apparent solubility profile for the repaglinide–HPβCD–L-arginine system and identifies the pH region where ternary interactions most effectively increase dissolved drug concentration.

### 2.3. Formulation-Relevant Descriptors

To interpret the calculated equilibria in terms relevant for pharmaceutical formulations, three complementary descriptors were introduced: the degree of precipitation, the synergistic coefficient, and the absolute solubility enhancement.

The degree of precipitation, $\gamma_{S}\left(pH\right)$, was defined as the fraction of total drug remaining in the solid phase at equilibrium:

$$\gamma_{S}\left(pH\right)=\left[solid\;drug\right]/C_{drug}^{0},$$

where $C_{drug}^{0}$ represents the total analytical drug concentration. Lower $\gamma_{S}$ values correspond to more efficient solubilization.

The synergistic coefficient, $SC\left(pH\right)$, was defined as:

$$SC\left(pH\right)=S_{ternary}(pH)/S_{binary}(pH)$$

where $S_{ternary}(pH)$ represents the apparent solubility in the drug–cyclodextrin–L-arginine system and $S_{binary}(pH)$ corresponds to the apparent solubility in the corresponding binary drug–cyclodextrin system under identical conditions. Values of $SC\left(pH\right)$ above unity indicate additional solubility enhancement associated with ternary interactions.

An additional descriptor of non-additive solubilization was introduced as:

$$∆S_{synergy}\left(pH\right)=S_{ternary}\left(pH\right)-S_{reference}\left(pH\right),$$

where $S_{reference}(pH)$ corresponds to the apparent solubility calculated under identical conditions in the absence of ternary association. This parameter isolates the direct contribution of multicomponent interactions to solubility enhancement. The detailed derivation of these descriptors from the coupled equilibrium equations is provided in Appendix A (Appendix A.4, Appendix A.5 and Appendix A.6).

**Figure 2:**
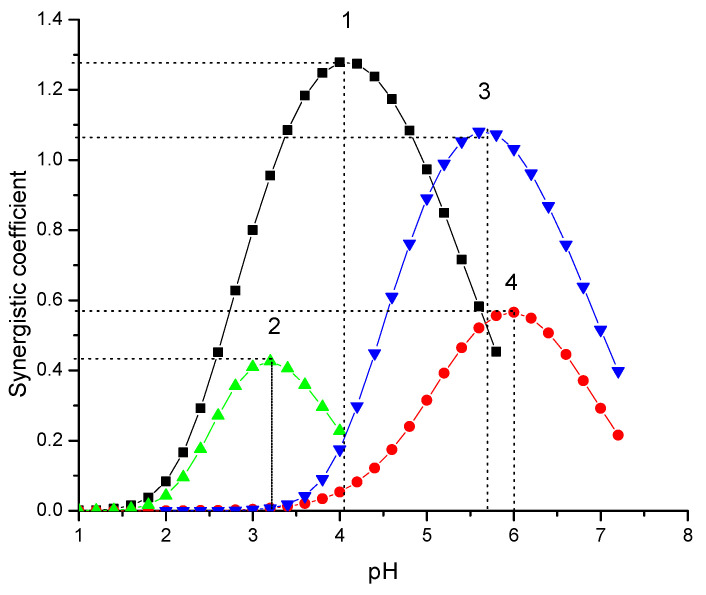
pH dependence of the synergistic coefficient (*SC*) in ternary drug–cyclodextrin–L-arginine systems at equimolar component concentrations (0.015 mol·L^−1^). Curves correspond to meloxicam (1), cefixime (2), repaglinide (3), and sulfadiazine (4). The profiles highlight drug-specific pH windows where ternary association most effectively enhances solubility, providing guidance for formulation design.

These descriptors provide complementary information about dissolved drug fraction, precipitation tendency, and multicomponent enhancement. The physical significance of $∆S_{synergy}\left(pH\right)$ is illustrated in Figure 1, which separates the contributions of pH-dependent ionization and ternary interactions to the overall solubility enhancement. Figure 2 shows the pH dependence of the synergistic coefficient for the investigated ternary systems and identifies formulation-relevant pH regions where additional solubility enhancement becomes significant.

The model was developed using standard assumptions of equilibrium solubility analysis, including saturation conditions, attainment of equilibrium, and dilute-solution behavior in the presence of a residual solid phase. The implications of these assumptions for predictive reliability and formulation analysis are discussed below.

### 2.4. Quantitative Validation Metrics

To provide a quantitative assessment of the agreement between calculated and published apparent solubilities, the relative error, mean relative error, mean absolute error, and root mean square error were calculated. For each point, the relative error was defined as:

$${RE}_{i}\left(\%\right)=\left|S_{calc,i}-S_{exp,i}\right|/S_{exp,i}\times 100$$

where $S_{calc,i}$ and $S_{exp,i}$ are the calculated and experimental apparent solubilities, respectively.

The mean relative error was calculated as:

$$MRE\left(\%\right)=\left(1/n\right)\sum_{i=1}^{n}\left|S_{calc,i}-S_{exp,i}\right|/S_{exp,i}\times 100$$

The mean absolute error was calculated as:

$$MAE=\left(1/n\right)\sum_{i=1}^{n}\left|S_{calc,i}-S_{exp,i}\right|$$

The root mean square error was calculated as:

$$RMSE={\left[\left(1/n\right)\sum_{i=1}^{n}{\left(S_{calc,i}-S_{exp,i}\right)}^{2}\right]}^{1/2}$$

These metrics were used to evaluate numerical agreement between the model and published phase-solubility data under comparable equilibrium conditions. When the stability constants were derived from the same phase-solubility dataset, the comparison was interpreted as a quantitative consistency check and not as fully independent external validation.

## 3. Results and Discussion

### 3.1. pH-Dependent Solubility Profiles

**Figure A1:**
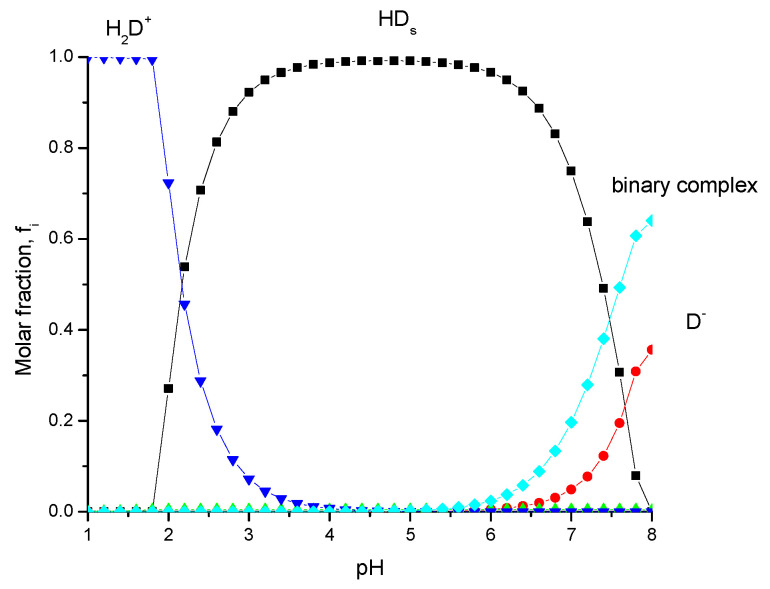
Diagrams of heterogeneous chemical equilibria for the binary *RPG*–*CD* system, calculated at total drug and cyclodextrin concentrations of 0.015 mol L^−1^.

**Figure A2:**
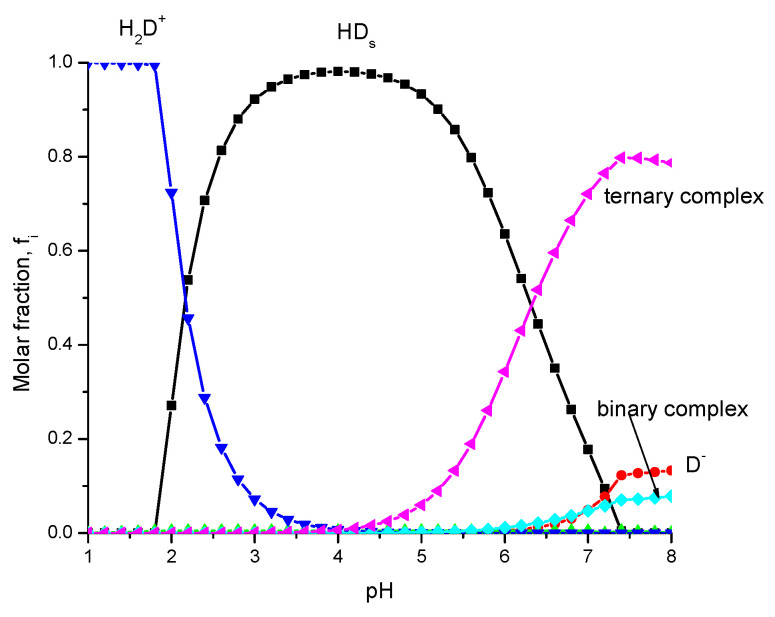
Diagrams of heterogeneous chemical equilibria for the ternary *RPG*–*CD*–*L*-*ARG* system, calculated at total drug, cyclodextrin and L-arginine concentrations of 0.015 mol L^−1^.

The calculated solubility profiles showed a strong dependence of dissolved drug concentration on pH for all investigated ternary systems. The corresponding redistribution of dissolved and solid species across the pH range is presented in Figure A1 and Figure A2 (Appendix C). Representative behavior for the repaglinide–HPβCD–L-arginine system is presented in Figure 1. The profiles demonstrate that the effect of cyclodextrin and L-arginine on apparent solubility is limited to specific pH regions where ionization and multicomponent interactions favor stabilization of dissolved drug species.

At acidic pH, the dissolved fraction remained relatively low because a substantial part of the drug persisted in the solid phase. Under these conditions, the contribution of ternary association to apparent solubility was limited. With increasing pH, the concentration of dissolved species increased sharply, reflecting changes in drug ionization and increasing participation of cyclodextrin and protonated arginine species in multicomponent equilibria. The resulting increase in apparent solubility was accompanied by a corresponding decrease in the residual solid fraction.

The calculations indicate that maximum solubility enhancement occurs within a relatively narrow pH interval. In this region, ionization equilibria and ternary interactions act simultaneously to stabilize dissolved drug species. Outside this interval, the dissolved concentration decreased because the equilibrium composition no longer favored formation of the dominant solubilizing species. Similar behavior was observed for sulfadiazine, cefixime, and meloxicam systems, although the position and width of the favorable pH region differed among the investigated drugs.

For systems where comparable experimental data were available, the calculated profiles reproduced the main solubility trends. For cefixime and meloxicam, the results should be interpreted as model-based predictions requiring further experimental confirmation [8,9,10,11]. In particular, the model reproduced the experimentally observed shift toward higher dissolved drug concentrations under conditions where ternary interactions become significant. The agreement between theoretical and experimental behavior supports the applicability of the equilibrium approach for analysis of multicomponent formulation systems.

Figure 1 also illustrates that the apparent solubility enhancement cannot be attributed solely to pH-induced ionization. In several pH regions, the increase in dissolved drug concentration exceeded the values expected from ionization effects alone, indicating an additional contribution associated with multicomponent interactions involving cyclodextrin and arginine species. This behavior became particularly pronounced near the pH region where the concentration of protonated arginine species remained sufficiently high to participate in ternary association processes.

The equilibrium redistribution of dissolved and solid species with pH is illustrated in Figure A1 and Figure A2 (Appendix C), which show the progressive replacement of the solid phase by binary and ternary dissolved complexes.

From a formulation perspective, the calculated profiles identify pH regions where dissolved drug concentrations can be maximized while minimizing the residual solid fraction. These results demonstrate the importance of simultaneous optimization of pH and excipient composition in cyclodextrin-based ternary formulations.

### 3.2. Influence of Cyclodextrin and L-Arginine on Apparent Solubility

Cyclodextrin increased apparent solubility in all investigated systems through formation of inclusion complexes with dissolved drug species. Addition of L-arginine produced a further increase in dissolved drug concentration, particularly within pH regions where ionized drug species and protonated arginine forms coexisted in solution.

The extent of solubility enhancement depended strongly on pH. At low pH values, the contribution of ternary association remained limited because the concentration of ionized drug species was low. Increasing pH shifted the equilibrium distribution toward species capable of participating more effectively in binary and ternary interactions, leading to higher apparent solubility.

The influence of L-arginine became most pronounced within restricted pH intervals corresponding to favorable overlap between drug ionization and arginine protonation. Outside these regions, the contribution of ternary stabilization decreased and the calculated profiles gradually approached the behavior of the corresponding binary drug–cyclodextrin systems.

The calculated values are compared with the available experimental solubility data in Table 2.

Table 2 provides a concise comparison with the literature data available under specified conditions. It is not intended as a full validation of all calculated profiles. A point-by-point quantitative check was possible only for the RPG–HPβCD–L-arginine phase-solubility dataset reported by Vakani et al. [8]. This analysis is given in Section 3.4 and Table A3 (Appendix B), where six concentration points reconstructed from the reported experimental regression are compared with the calculated values across the HPβCD concentration range used in the source study.

For repaglinide, the calculated apparent solubility at pH 5.8 was close to the experimentally reported ternary-system solubility, although the pH value was not reported in the original solubility study. A similar level of agreement was obtained for sulfadiazine at alkaline pH. For cefixime and meloxicam, the calculated apparent solubilities at the pH values corresponding to complete dissolution predicted by the model were added to Table 2. These values indicate the predicted upper dissolved drug concentration under the imposed total drug concentration of 0.015 mol L^−1^. The strongest relative ternary contribution, expressed by the synergistic coefficient, occurred at somewhat lower pH values; however, at these points a residual solid fraction was still present. Therefore, the values reported in Table 2 were selected according to the combined criterion of high apparent solubility and minimal residual solid fraction. The quantitative validation metrics for the RPG–HPβCD–L-arginine phase-solubility comparison are reported in Section 3.4.

Figure 2 shows the pH dependence of the synergistic coefficient for the investigated drugs. The profiles reveal distinct pH regions where ternary association contributes most effectively to solubility enhancement. Repaglinide and sulfadiazine exhibit broader regions of positive enhancement extending toward near-neutral pH, whereas cefixime and meloxicam display narrower acidic domains. The position of these maxima reflects the combined influence of drug ionization, cyclodextrin complexation, and arginine speciation.

The obtained results show that the influence of cyclodextrin and L-arginine cannot be considered separately because both components affect the same coupled equilibrium system. Changes in pH simultaneously modify ionization state, complex stability, and distribution between dissolved and solid phases. From a formulation perspective, these relationships are important for identifying pH regions where dissolved drug concentration can be increased while minimizing precipitation.

### 3.3. Precipitation Behavior and Residual Solid Fraction

**Figure 3:**
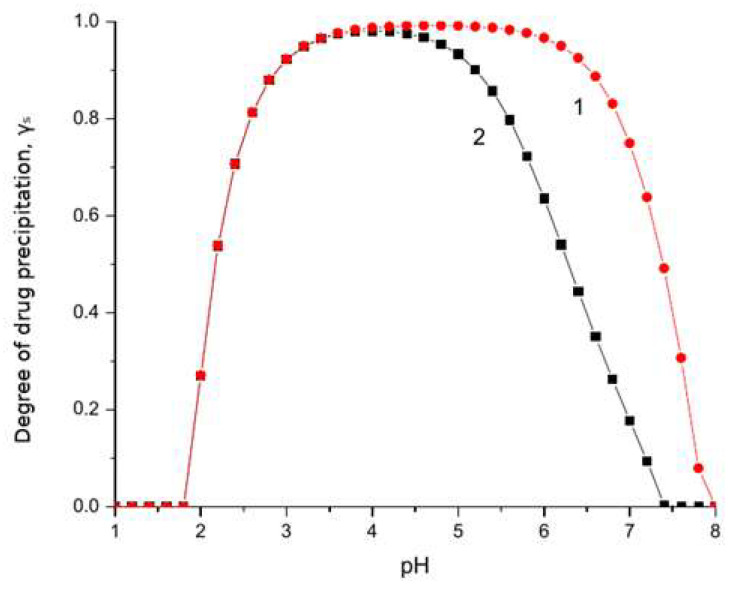
Computed degree of drug precipitation as a function of pH for the binary *RPG*–*CD* system (curve 1) and ternary *RPG*–*CD*–*L*-arginine system (curve 2) at $C_{D}^{0}=C_{CD}^{0}=C_{A}^{0}=$ 0.015 mol L^−1^.

The calculated precipitation profiles showed that a considerable fraction of the drug remained in the solid phase outside favorable solubilization regions. Figure 3 presents the pH dependence of the residual solid fraction for binary and ternary repaglinide systems. In the presence of L-arginine, the amount of undissolved drug decreased over the pH interval where ternary interactions contributed to stabilization of dissolved species.

At acidic pH, the dissolved drug concentration remained low and most of the drug persisted in the solid phase. Increasing pH shifted the equilibrium toward dissolved forms and progressively reduced the residual solid fraction. The transition occurred over a broader pH interval in the ternary systems than in the corresponding binary systems, indicating improved stabilization of dissolved drug species in the presence of L-arginine.

The decrease in precipitation coincided with the pH region where the synergistic coefficient reached its highest values (Figure 2). Under these conditions, ternary association increased the fraction of dissolved species and reduced the amount of residual solid drug more effectively than binary cyclodextrin complexation alone.

Differences among the investigated drugs were also evident. Repaglinide and sulfadiazine retained relatively broad pH regions where the dissolved fraction remained high, whereas cefixime and meloxicam exhibited narrower domains of effective stabilization. Outside these intervals, the fraction of undissolved drug increased rapidly because the equilibrium composition no longer favored stabilization of dissolved species.

For RPG and SDZ, the calculated trends were consistent with the available experimental observations summarized in Table 2. For CEF and MLX, the calculated profiles are presented as predictive outputs requiring further experimental confirmation. The calculations reproduced the experimentally observed relationship between increased apparent solubility and reduced precipitation in ternary cyclodextrin systems. These results indicate that precipitation suppression and solubility enhancement originate from the same coupled equilibrium processes controlling distribution between dissolved and solid phases.

From a formulation standpoint, the calculated precipitation profiles are relevant for predicting changes that may occur after dilution or transfer into media with different pH values. A formulation showing high apparent solubility under one set of conditions may undergo rapid precipitation after pH change. The calculated profiles therefore help identify pH regions where dissolved drug concentrations remain high while the residual solid fraction stays low.

### 3.4. Quantitative Comparison with Published Phase-Solubility Data

A quantitative comparison was performed for the RPG–HPβCD–L-arginine system using the phase-solubility data reported by Vakani et al. [8]. This dataset was selected because it represents an equilibrium phase-solubility experiment performed at defined HPβCD concentrations. In contrast, saturation solubility values obtained for physical mixtures, kneaded products, or co-evaporated products also reflect solid-state preparation effects and were therefore used only for qualitative comparison.

The experimental apparent solubilities were reconstructed from the regression equation reported for the ternary RPG–HPβCD–L-arginine system [8]:

$$S_{exp}\left(mM\right)=0.2541C_{HP\beta CD}\left(mM\right)+3.6002$$

where $C_{HP\beta CD}$ is the analytical concentration of HPβCD in mM. The comparison was performed at $C_{HP\beta CD}$ = 0, 3, 6, 9, 12, and 15 mM. The calculated slope was obtained from the phase-solubility expression:

$${slope}_{calc}=K_{s}S_{0}/\left(1+K_{s}S_{0}\right)$$

using $K_{s}$ = 4407.01 M^−1^ and $S_{0}$ = 7.73 × 10−5 mol L^−1^. This gave ${slope}_{calc}$ = 0.2540998, which is practically identical to the published experimental slope of 0.2541.

The detailed point-by-point comparison between the reconstructed experimental values and the calculated values is provided in Table A3 (Appendix B). The calculated values reproduced the published phase-solubility data for the repaglinide–HPβCD–L-arginine system [8], with MRE = 2.77 × 10^−5^%, MAE = 1.73 × 10^−9^ mol L^−1^, and RMSE = 2.09 × 10^−9^ mol L^−1^. These very small deviations arise because the comparison uses the regression equation and stability constant reported for the same phase-solubility dataset. The purpose of this analysis is to verify numerical consistency of the mass-balance implementation, while broader predictive performance must be assessed with independent datasets. Therefore, this comparison should be interpreted as a quantitative consistency check and not as fully independent external validation. Broader validation will require additional datasets measured under comparable pH, ionic strength, and excipient concentration conditions.

### 3.5. Comparison with Literature Data and Predictive Applications

The quantitative comparison presented above confirms that the equilibrium model reproduces the published phase-solubility behavior of the RPG–HPβCD–L-arginine system [8] when the same stability constant and concentration range are used. This result supports the internal consistency of the mass-balance treatment and confirms that the model correctly translates the reported association constant into pH-dependent apparent solubility profiles.

For additional comparison, the calculated apparent solubility of the RPG–HPβCD–L-arginine system at pH 5.8 was 0.042 mol L^−1^, which is close to the reported experimental value near pH 6.0 of approximately 0.040 mol L^−1^ [8]. A similar level of agreement was obtained for sulfadiazine under alkaline conditions, where the calculated value of 0.048 mol L^−1^ was close to the reported experimental solubility of 0.052 mol L^−1^ [9]. These comparisons indicate that the coupled equilibrium approach captures the principal physicochemical factors controlling solubility enhancement in the validated systems.

For cefixime [10,26] and meloxicam [11,27,28], directly comparable experimental apparent-solubility datasets under the same cyclodextrin–L-arginine conditions were not available. These systems were therefore treated as predictive applications of the equilibrium framework. The calculated profiles indicate that both systems exhibit narrower pH domains in which ternary stabilization is effective. Outside these domains, the residual solid fraction remains substantial despite the presence of cyclodextrin and L-arginine.

The model also reproduced the restriction of enhanced solubilization to defined pH intervals. In the investigated systems, ternary stabilization became more pronounced only within regions where favorable overlap existed between drug ionization and arginine protonation. Outside these intervals, the calculated dissolved fraction decreased and the residual solid fraction increased. This behavior shows that pH adjustment alone cannot fully explain the observed enhancement of apparent solubility. The largest increases occurred when ionization, cyclodextrin complexation, and ternary association contributed simultaneously to stabilization of dissolved drug species.

### 3.6. Mechanistic Basis of Ternary Complexation

The calculated increase in apparent solubility in the ternary systems is related to the simultaneous presence of ionized drug species, cyclodextrin, and protonated L-arginine species in solution. In the present model, ternary association was described mainly through complexes involving the anionic drug form, cyclodextrin, and H_2_A^+^ as the relevant protonated form of L-arginine. This choice follows the equilibrium constants reported in the literature sources used for the calculations. At moderate pH, other arginine protonation states may also be present. Their quantitative contribution can be included only when the corresponding independent association constants are available. In the present calculations, the dominant ternary term was restricted to the species supported by the literature constants used in Table 1.

The role of L-arginine can be explained by several physicochemical interactions. The protonated guanidinium group of arginine may interact with ionized acidic drug species through electrostatic attraction and ion-pair formation. Hydrogen bonding may also occur between polar groups of the drug, arginine, and the hydroxyl groups located at the outer surface of cyclodextrin. At the same time, the hydrophobic region of the drug molecule can be included in the cyclodextrin cavity, while the charged or polar groups remain exposed to the aqueous phase. Thus, cyclodextrin inclusion and arginine association can jointly stabilize dissolved drug-containing species.

This interpretation is consistent with experimental reports showing that L-arginine increases apparent stability constants, complexation efficiency, and apparent solubility in ternary cyclodextrin systems. However, the present model does not provide direct structural proof of a unique ternary complex geometry. Direct molecular-level confirmation would require additional evidence from techniques such as 2D NMR, ROESY/NOESY, FTIR under comparable conditions, or molecular dynamics simulations. The present treatment describes the equilibrium consequences of ternary association using available constants.

Higher-order complexes, including 2:1 cyclodextrin–drug species, may occur for large molecules such as repaglinide, especially at high cyclodextrin concentrations or when phase-solubility diagrams deviate from AL-type behavior. They were not included in the present calculations because the analyzed literature dataset reported AL-type behavior and 1:1 stoichiometry for the relevant RPG–HPβCD systems. Introducing 2:1 species without reliable constants would add additional terms not supported by the available data. The same mass-balance framework can be extended to include 2:1 or other higher-order species when reliable equilibrium constants are available.

### 3.7. Practical Formulation Implications

From a formulation perspective, the calculated profiles provide a basis for identifying pH regions where solubility enhancement and precipitation suppression occur simultaneously. These regions are important because a formulation optimized under one set of conditions may lose dissolved drug after dilution or transfer into a medium with a different pH. This situation is especially relevant for oral formulations, where the drug may experience rapid transitions between acidic gastric conditions and near-neutral or weakly alkaline intestinal environments.

For oral delivery, the pH dependence of these systems is especially important. A formulation may first be exposed to acidic gastric fluid and then to near-neutral intestinal fluid. During this transition, the ionization state of the drug and the protonation state of L-arginine change at the same time. As a result, a formulation that maintains a high dissolved drug concentration in one pH region may become vulnerable to precipitation after transfer to another pH region. The calculated solubility and residual solid profiles identify these vulnerable pH intervals and can help select formulation conditions with a lower risk of precipitation after pH transition.

The present results suggest that cyclodextrin and L-arginine should not be optimized independently. Their combined effect depends on the pH-dependent availability of the ionized drug form, free cyclodextrin, and protonated arginine species participating in ternary association. Therefore, the most favorable formulation region is not necessarily the pH at which the free drug alone is most soluble. It is the pH interval where drug ionization, inclusion complexation, and ternary stabilization act together.

The model can be used as a preliminary screening tool to select formulation pH, cyclodextrin concentration, and L-arginine concentration before experimental optimization. Conditions predicted to give high apparent solubility together with a low residual solid fraction should be prioritized experimentally. In contrast, pH regions where the calculated ternary complex fraction decreases and the residual solid fraction increases should be avoided when precipitation suppression is required.

These recommendations should be applied within the applicability domain of the model. Final formulation selection still requires experimental confirmation under the intended dosage-form conditions, especially when ionic strength, buffer composition, excipient concentration, or solid-state form differs from the conditions used to derive the equilibrium constants.

### 3.8. Applicability Domain and Limitations

The proposed model is applicable to saturated drug–cyclodextrin–L-arginine systems where apparent solubility is controlled by acid–base equilibria, inclusion complexation, ternary association, and distribution between dissolved species and a residual solid drug phase. Its use requires reliable equilibrium constants for drug ionization, cyclodextrin complexation, arginine protonation, and the dominant ternary association reactions. Nanoparticle or colloidal association of cyclodextrin complexes was not included as a separate equilibrium in the present calculations. The model describes molecular speciation, including acid–base equilibria, binary inclusion complexation, cyclodextrin–arginine association, ternary association, and distribution between dissolved species and the residual solid drug phase. Aggregation would require additional parameters, including aggregation constants, aggregate-size distributions, and their dependence on pH, ionic strength, cyclodextrin concentration, and drug loading. For systems where pH-dependent cyclodextrin aggregates or nanoparticles make a substantial contribution to apparent solubility, additional experimental characterization and an extended aggregation model would be required.

The present validation is strongest for the RPG–HPβCD–L-arginine system, where published phase-solubility data were available over a defined cyclodextrin concentration range [8]. Additional comparison was possible for sulfadiazine [9]. For cefixime [10,26] and meloxicam [11,27,28], directly comparable apparent-solubility datasets under the same cyclodextrin–L-arginine conditions were not available. These systems should therefore be regarded as predictive applications of the same equilibrium framework, not as independently validated cases.

The current examples mainly concern poorly soluble ionizable drugs with acidic or amphoteric behavior. Extension to weakly basic, neutral, or other amphoteric drugs is possible, but it requires the corresponding ionization constants, cyclodextrin binding constants, and ternary association constants. The mathematical form of the model can accommodate weak bases, but reliable numerical prediction depends on the availability and quality of the relevant constants.

The calculations refer to L-arginine because the equilibrium constants used in the model were reported for L-arginine-containing systems. Replacement by D-arginine or racemic arginine would require independent association constants, since cyclodextrin cavities and several drug molecules are chiral environments. The stereochemical form of the drug was taken as reported in the original experimental sources. No separate enantiomer-specific constants were introduced.

The model assumes equilibrium conditions and dilute-solution behavior. It does not describe nucleation, crystal growth, precipitation kinetics, metastable supersaturation, or changes in solid-state form during preparation. Ionic strength and buffer composition may also influence protonation and association constants. Therefore, predictions made outside the experimental conditions used to derive the constants should be interpreted with caution.

Within these limits, the model can be used as a screening tool for identifying pH regions and excipient compositions where solubility enhancement and precipitation suppression are expected to occur together. Final formulation selection still requires experimental confirmation under the intended dosage-form conditions.

## 4. Conclusions

The present study demonstrates that pH-dependent solubility enhancement in drug–cyclodextrin–L-arginine systems is controlled by coupled equilibria involving ionization, binary inclusion complexation, ternary association, and distribution between dissolved and solid phases. The profiles revealed that enhanced apparent solubility and reduced precipitation occur only within limited pH regions where these processes act simultaneously.

Quantitative comparison with published phase-solubility data confirmed the numerical consistency of the model for the RPG–HPβCD–L-arginine system. The additional systems illustrate predictive applications of the same equilibrium framework, but supplementary experimental validation is required under directly comparable formulation conditions.

The data suggest that the effect of L-arginine depends strongly on pH and becomes most pronounced within regions where protonated arginine species coexist with drug forms capable of participating in multicomponent association. Outside these regions, the systems gradually approach the behavior of the corresponding binary drug–cyclodextrin formulations.

The calculations also showed that apparent solubility enhancement cannot be explained solely by pH-induced ionization. Additional stabilization arises from ternary interactions involving cyclodextrin complexes and protonated arginine species. The extent of this effect varies among different drugs according to their acid–base properties and complex stability.

From a formulation perspective, the approach provides a useful basis for identifying pH regions associated with increased dissolved drug concentration and reduced precipitation tendency. It may support preliminary optimization of formulation pH and excipient composition while reducing the extent of empirical screening during the development of cyclodextrin-containing formulations. The reliability of predictions outside the investigated conditions will depend on the availability of equilibrium constants measured under comparable pH, ionic strength, buffer composition, and excipient concentration conditions.

## Figures and Tables

**Table 1:** The equilibrium constants of the considered reactions at 25 °C in the analyzed systems.

Species	Equilibrium	logK	Constants Notation	Source
HPβCD (CD)-L-arginine ($H_{2}A^{+}$)	$CD+H_{2}A^{+}=CD\cdot H_{2}A^{+}$	1.77	$K_{CD:H_{2}A^{+}}$	[14]
Repaglinide (*RPG*)-HPβCD	$RPG^{-}+CD=\left[RPG\cdot CD^{-}\right]$	2.52	$K_{1:1}$	[8]
Repaglinide-HPβCD∙L-arginine	$\left[RPG\cdot CD^{-}\right]+H_{2}A^{+}=\left[RPG\cdot CD^{-}\cdot H_{2}A^{+}\right]$ $RPG^{-}+CD+H_{2}A^{+}=\left[RPG\cdot CD^{-}\cdot H_{2}A^{+}\right]$	3.65 6.17	$K_{tern}$ $\beta_{111}$	[8]
L-arginine	$A^{-}+H^{+}=HA$ $A^{-}+2H^{+}=H_{2}A^{+}$ $A^{-}+3H^{+}=H_{3}A^{2+}$	10.13 18.66 21.47	$\beta_{1}$ $\beta_{2}$ $\beta_{3}$	[12] [12,13] [12,13]
Repaglinide	$H_{2}{RPG}^{+}=HRPG+H^{+}$ $HRPG⇌{RPG}^{-}+H^{+}$	4.2 6.0	$K_{a,1}$ $K_{a,2}$	[8]
Sulfadiazine (*SDZ*)-βCD (*CD*)	${SDZ}^{-}+CD=\left[SDZ\cdot CD^{-}\right]$	2.45	$K_{1:1}$	[9]
Sulfadiazine-βCD∙L-arginine	$\left[SDZ\cdot CD^{-}\right]+H_{2}A^{+}=\left[SDZ\cdot CD^{-}\cdot H_{2}A^{+}\right]$ ${SDZ}^{-}+CD+H_{2}A^{+}=\left[SDZ\cdot CD^{-}\cdot H_{2}A^{+}\right]$	3.06 5.51	$K_{tern}$ $\beta_{111}$	[9]
Sulfadiazine	$H_{2}{SDZ}^{+}=HSDZ+H^{+}$ $HSDZ⇌{SDZ}^{-}+H^{+}$	2.2 6.3	$K_{a,1}$ $K_{a,2}$	[21]
Cefixime (*CEF*)-HPβCD (*CD*)	${CEF}^{-}+CD=\left[CEF\cdot CD^{-}\right]$	2.64	$K_{1:1}$	[10]
Cefixime-HPβCD∙L-arginine	$\left[CEF\cdot CD^{-}\right]+H_{2}A^{+}=\left[CEF\cdot CD^{-}\cdot H_{2}A^{+}\right]$ ${CEF}^{-}+CD+H_{2}A^{+}=\left[CEF\cdot CD^{-}\cdot H_{2}A^{+}\right]$	3.10 5.74	$K_{tern}$ $\beta_{111}$	[10]
Cefixime	$H_{2}{CEF}^{+}=HCEF+H^{+}$ $HCEF⇌{CEF}^{-}+H^{+}$	1.89 3.80	$K_{a,1}$ $K_{a,2}$	[22]
Meloxicam (*MLX*)-HPβCD (*CD*)	${MLX}^{-}+CD=\left[MLX\cdot CD^{-}\right]$	2.36	$K_{1:1}$	[11]
Meloxicam-HPβCD∙L-arginine	$\left[MLX\cdot CD^{-}\right]+H_{2}A^{+}=\left[MLX\cdot CD^{-}\cdot H_{2}A^{+}\right]$ ${MLX}^{-}+CD+H_{2}A^{+}=\left[MLX\cdot CD^{-}\cdot H_{2}A^{+}\right]$	3.84 6.20	$K_{tern}$ $\beta_{111}$	[11]
Meloxicam	$H_{2}{MLX}^{+}=HMLX+H^{+}$ $HMLX⇌{MLX}^{-}+H^{+}$	1.1 4.2	$K_{a,1}$ $K_{a,2}$	[23,24,25]

**Table 2:** Comparison between experimentally reported and calculated apparent solubilities for representative drug–cyclodextrin–arginine systems under specified conditions.

Drug	Intrinsic Solubility, $S_{0}$, mol·L^−1^	Experimental Apparent Solubility at Specific Conditions, $S_{app,tern}$, mol·L^−1^	Calculated Apparent Solubility at Specific Conditions, $S_{app,tern}$, mol·L^−1^	Sources and Additional Information
RPG	7.7 × 10^−5^	0.040; 25 °C ^a^, pH not reported ^b^	0.042, 25 °C, pH 5.8	*S*_0_, *S*_*app*,*tern*_ [8]
SDZ	2.9 × 10^−4^	0.052; 25 °C ^a^, pH 8.0	0.048, 25 °C, pH 8.0	*S*_0_, *S*_*app*,*tern*_ [9]
CEF	1.7 × 10^−3^	Related ternary solubilization data reported ^c^	0.015, 25 °C, pH 3.8 ^d^	[10,26]
*MLX*	3.4 × 10^−5^	Related ternary solubilization data reported ^e^	0.015, 25 °C, pH 6.0 ^f^	[11,27,28]

^a^ Apparent solubilities reported under different experimental conditions reflect the original literature sources. ^b^ The pH value was not reported in the original repaglinide solubility study; therefore, the calculated value at pH 5.8 is used only as an approximate comparison under near-neutral conditions. ^c^ Ternary cefixime–HPβCD–amino acid/cyclodextrin solubilization was reported experimentally, but no directly comparable pH-dependent apparent-solubility dataset under the same equilibrium conditions was available. ^d^ Calculated predictive value at the pH where complete dissolution predicted by the model is reached; related experimental solubilization data are reported in Refs. [10,26]. ^e^ Related ternary meloxicam–HPβCD–L-arginine solubilization/dissolution data were reported, but no directly comparable pH-dependent equilibrium apparent-solubility dataset was available. ^f^ Calculated predictive value at the pH where complete dissolution predicted by the model is reached; related experimental ternary-complex data are reported in Refs. [11,27,28]. For CEF and MLX, the calculated values correspond to the pH at which the model predicts complete dissolution of the total drug amount used in the calculations, 0.015 mol L^−1^. Therefore, these values are presented as predictive model outputs and should not be interpreted as independent experimental validation.

## Data Availability

The original contributions presented in this study are included in the article and Appendix A, Appendix B and Appendix C. Further inquiries can be directed to the corresponding author(s).

## Appendix A. Expressions Derived from the Thermodynamic Model

### Appendix A.1. Protonation Equilibria Governing Drug Solubility

The quantity $\left[D^{-}\right]$ means the equilibrium concentration of the ionized form, which depends on pH via the Henderson-Hasselbalch equation:

(A1) $$\left[D^{-}\right]=S_{0}\cdot 10^{pH-pK_{a}}.$$

The acid dissociation constant of the drug $K_{a}$ is calculated by the expression:

(A2) $$HD=H^{+}+D^{-},\;K_{a}=\left[D^{-}\right][H^{+}]/\left[HD\right]$$

The fraction of ionized versus neutral form depends on the Henderson-Hasselbalch equation:

(A3) $$\left[D^{-}\right]/\left[HD\right]=10^{pH-pK_{a}}$$

Similarly, for a weak base (as benzydamine):

(A4) $$D_{\left(S\right)}=D,\;K_{D}^{\left(S\right)}=\left[D\right]/\left[D_{S}\right]=\left[D\right]=S_{0}$$

(A5) $$S_{app}=\left[D\right]+[{DH}^{+}]$$

or

(A6) $$S_{app}=S_{0}\cdot {(1+10}^{pK_{a}-pH}).$$

(A7) $$D+H^{+}={DH}^{+},K_{a}=\left[{DH}^{+}\right]/\left(\left[D][H^{+}\right]\right).$$

In this case, the fraction of ionized form to neutral one is:

(A8) $$\frac{\left[{DH}^{+}\right]}{\left[D\right]}=10^{pK_{a}-pH}$$

Diprotic drugs such as repaglinide can be described by two consecutive protonation equilibria within the relevant pH range:

(A9) $$H_{2}D^{+}=HD+H^{+},\;K_{a,1}=\left[HD\right][H^{+}]/\left[H_{2}D^{+}\right]$$

(A10) $$HD⇌D^{-}+H^{+},\;K_{a,2}=\left[D^{-}\right][H^{+}]/\left[HD\right]$$

For a weakly acidic drug, its speciation is pH-dependent and transitions across protonated, neutral, and ionized forms depending on pH. In the absence of cyclodextrin *CD* and an auxiliary agent *HA*, the total solubility $S_{app}$ is given by the expression:

(A11) $$S_{app}=[HD]+{\left[D^{-}\right]+\left[H_{2}D^{+}\right]=S}_{0}\left(1+K_{a1}^{-1}\left[H^{+}\right]+K_{a2}[H^{+}{]}^{-1}\right)$$

The pH-dependent distribution of drug species defines the speciation domain for binary and ternary complexation.

### Appendix A.2. Protonation Equilibria of the Auxiliary Agent

L-arginine participates in complexation through its protonated forms. Its triprotic protonation behavior is described by the following equilibria:

(A12) $$A^{-}+H^{+}=HA,\;\beta_{1}=\left[HA\right]/\left(\left[A^{-}\right][H^{+}]\right)$$

(A13) $$A^{-}+{2H}^{+}=H_{2}A^{+},\;\beta_{2}=\left[H_{2}A^{+}\right]/\left(\left[A^{-}\right][H^{+}{]}^{2}\right)$$

(A14) $$A^{-}+{3H}^{+}=H_{3}A^{2+},\;\beta_{3}=\left[H_{3}A^{2+}\right]/\left(\left[A^{-}\right][H^{+}{]}^{3}\right)$$

The overall protonation constants $\beta_{i}$ determine the distribution of L-arginine species that are available for binary and ternary complex formation. These constants govern the distribution of $A^{-}$, HA, $H_{2}A^{+}$ and $H_{3}A^{2+}$ across the pH range relevant for pharmaceutical formulations.

### Appendix A.3. Ternary Association Equilibria

Considering the corresponding ternary association equilibria described in the main text, the overall formation constant $\beta_{111}$ is expressed as:

(A15) $$\beta_{111}\;=\;K_{1:1}\cdot K_{tern}=[HD\cdot CD\cdot HA]\;/\;([HD][CD\;][HA])$$

(A16) $$\beta_{111}=K_{1:1}\cdot K_{tern}=[D^{-}\cdot CD\cdot H_{2}A^{+}]\;/\;([D^{-}][CD][H_{2}A^{+}])$$

For different protonation domains, ternary association may be written in a general form; in the calculations reported here, the dominant ternary term was represented by Equation (A16). These equilibria describe the supplementary stabilization that is responsible for the cooperative increase in drug solubility in ternary systems.

### Appendix A.4. Thermodynamic Representation of the Heterogeneous System

For drugs belonging to the analyzed class of weakly acidic, poorly soluble compounds, the predominant species under physiological pH conditions depends on the specific $pK_{a}$ values and pH, and may include neutral and ionized forms; therefore, no single species predominance is assumed across the entire pH range, and speciation must be evaluated within defined pH domains.

The mass balance (*MB*) equations, taking into account both phases, are formulated by accounting for the interactions of the drug (*HD*) with cyclodextrin (*CD*) and auxiliary agent (Arg) (*HA*), including the formation of binary ($D^{-}\cdot CD$) and ternary ($D^{-}\cdot CD\cdot H_{2}A^{+}$) complexes, as described in the paper:

(A17) $$C_{D}^{0}=\left[HD_{s}\right]+\left[D^{-}\right]+\left[HD\right]+\left[\;H_{2}D^{+}\right]+\left[D^{-}\cdot CD\right]+\left[D^{-}\cdot CD\cdot H_{2}A^{+}\right],$$

(A18) $$C_{CD}^{0}=\left[CD\right]+\left[D^{-}\cdot CD\right]+\left[CD\cdot H_{2}A^{+}\right]+[D^{-}\cdot CD\cdot H_{2}A^{+}]$$

(A19) $$C_{A}^{0}=\left[A^{-}\right]+\left[HA\right]+\left[\;H_{2}A^{+}\right]+\left[\;H_{3}A^{2+}\right]+\left[CD\cdot H_{2}A^{+}\right]+\left[D^{-}\cdot CD\cdot H_{2}A^{+}\right]$$

In Equation (A17), the term $\left[HD_{s}\right]$ signifies the number of moles of the precipitated drug per liter of aqueous saturated solution, essentially representing the molar concentration. This differs from $[HD_{\left(S\right)}$] in the paper main text, which refers to the concentration of the solid in its own phase and is conventionally set to unity. In Equations (A17)–(A19), $C_{i}^{0}$ denotes the total concentrations of component “*i*” in the heterogeneous system. Using the corresponding equilibrium constants, these concentrations can be expressed as:

(A20) $$C_{D}^{0}=\left[HD_{s}\right]+\left[HD\right]\left(1+K_{a1}^{-1}[H^{+}]+K_{a2}[H^{+}{]}^{-1}+K_{1:1}K_{a2}[H^{+}{]}^{-1}\left[CD\right]+{\beta_{111}K}_{a2}[H^{+}{]}^{-1}\left[CD\right][H_{2}A^{+}]\right)$$

(A21) $$C_{CD}^{0}=\left[CD\right](1+K_{1:1}K_{a2}[H^{+}{]}^{-1}\left[HD\right]+K_{CD:H_{2}A^{+}}\left[H_{2}A^{+}\right]+\beta_{111}K_{a2}[H^{+}{]}^{-1}\left[HD\right]\left[H_{2}A^{+}\right])$$

(A22) $$C_{A}^{0}=\left[H_{2}A^{+}\right](1+\beta_{2}^{-1}[H^{+}{]}^{-2}+{\beta_{1}\beta }_{2}^{-1}[H^{+}{]}^{-1}+{\beta_{3}\beta }_{2}^{-1}\left[H^{+}\right]+K_{CD:H_{2}A^{+}}\left[CD\right]+\beta_{111}K_{a2}[H^{+}{]}^{-1}\left[HD\right]\left[CD\right])$$

Given that $\left[HD\right]=S_{0}$, Equation (A20) can be reformulated to express the key *MB* equation for the drug as follows:

(A23) $$C_{D}^{0}=\left[HD_{s}\right]+S_{0}\left(1+K_{a1}^{-1}[H^{+}]+K_{a2}[H^{+}{]}^{-1}+K_{1:1}K_{a2}[H^{+}{]}^{-1}\left[CD\right]+\beta_{111}K_{a2}[H^{+}{]}^{-1}\left[CD\right][H_{2}A^{+}]\right)$$

Equation (A23) yields the following expression for the apparent ternary system solubility $S_{app,\;tern}$:

(A24) $$S_{app,\;tern}=S_{0}\left(1+K_{a1}^{-1}[H^{+}]+K_{a2}[H^{+}{]}^{-1}+K_{1:1}K_{a2}[H^{+}{]}^{-1}\left[CD\right]+\beta_{111}K_{a2}[H^{+}{]}^{-1}\left[CD\right][H_{2}A^{+}]\right)$$

No separate binary drug–arginine complex was included in this expression because a reliable independent association constant for $D^{-}-H_{2}A^{+}$ was not available for the analyzed systems. The effect of L-arginine was therefore represented through the experimentally reported $CD-H_{2}A^{+}$ and ternary $drug-CD-H_{2}A^{+}$ association terms. A separate *CD*–*HA* term was not included, since an independent association constant for this species was not available under comparable conditions. The mass-balance equations can be expanded to include this interaction once suitable experimental constants are reported.

Analogously, the apparent binary system solubility $S_{app,\;bin}$ is calculated by the expression:

(A25) $$S_{app,\;bin}=S_{0}\left(1+K_{a1}^{-1}[H^{+}]+K_{a2}[H^{+}{]}^{-1}+K_{1:1}K_{a2}[H^{+}{]}^{-1}\left[CD\right]\right)$$

In the main text, these quantities are denoted as $S_{app,\;tern}$ and $S_{app,\;bin}$, respectively, to emphasize their interpretation as apparent solubilities under defined conditions. The reference solubility $S_{reference}\left(pH\right)$ used in the main text is obtained from the same mass balance framework by setting the ternary formation constant to zero ${(\beta }_{tern}=0$), while preserving all other equilibrium relationships.

Provided that the initial chemical composition, $C_{D}^{0}$, $C_{CD}^{0}$, and $C_{A}^{0},$ for the heterogeneous system is known and with $\left[HD\right]=S_{0}$, the values of the variables $\left[HD_{s}\right],\left[CD\right]$ and $\left[H_{2}A^{+}\right]$ can be computed numerically through the solution of Equations (A20)–(A22), using standard numerical solvers. Equations (A23) and (A24) define the relationship between the total drug concentration $C_{D}^{0}$ and the total solubility $S_{app,\;tern}$. Together with the equilibrium constants introduced above, these mass balance expressions (A20)–(A24) form the basis for a complete equilibrium description of the heterogeneous system as a function of pH and composition. The solubility expressions derived in Equations (A24) and (A25) form the basis for the calculation of the synergistic descriptors *SC*(pH) and $∆S_{synergy}(pH)$ introduced in the main text. The descriptor $∆S_{synergy}(pH)$ is directly obtained as the difference between the solubilities defined in Equations (A24) and (A25), ensuring full consistency between the model and its interpretation.

### Appendix A.5. Distribution Profiles of Drug-Containing Species

The distribution profiles of drug-containing species in both aqueous and solid phases provide a clear depiction of the heterogeneous chemical equilibrium. The molar fraction expressions for each drug-containing species are given by:

$$f_{S}=\left[HD_{s}\right]/C_{D}^{0},\;{\;f}_{H_{2}D^{+}}=\left[\;H_{2}D^{+}\right]/C_{D}^{0}=S_{0}K_{a1}^{-1}[H^{+}]/C_{D}^{0},\;f_{HD}=\left[HD\right]/C_{D}^{0}=S_{0}/C_{D}^{0},$$

$$f_{D^{-}}=\left[D^{-}\right]/C_{D}^{0}=S_{0}K_{a2}[H^{+}{]}^{-1}/C_{D}^{0},\;f_{D^{-}-CD}=\left[D^{-}\cdot CD\right]/C_{D}^{0}=S_{0}K_{1:1}K_{a2}[H^{+}{]}^{-1}\left[CD\right]/C_{D}^{0},$$

(A26) $$f_{D^{-}\cdot CD\cdot H_{2}A^{+}}=\left[D^{-}\cdot CD\cdot H_{2}A^{+}\right]/C_{D}^{0}=S_{0}\beta_{111}K_{a2}[H^{+}{]}^{-1}\left[CD\right][H_{2}A^{+}]/C_{D}^{0}$$

From the expressions (A26) the following identity results:

(A27) $$f_{S}+f_{D^{-}}+f_{HD}+f_{H_{2}D^{+}}+f_{D^{-}\cdot CD}+f_{D^{-}\cdot CD\cdot H_{2}A^{+}}=1$$

For constructing a complete *DHCE* for an aqueous homogeneous solution, where solving Equations (A20)–(A22) results in $\left[HD_{s}\right]<\;0$, the molar fractions of solute species are calculated using the standard equations commonly applied in conventional species distribution diagrams [29].

### Appendix A.6. pH Region Corresponding to the SC Maximum

The synergistic coefficient *SC*(pH) reaches elevated values within the pH region where the concentrations of the anionic drug form $D^{-}$ and the diprotonated arginine species $H_{2}A^{+}$ become sufficiently high to favor formation of the ternary complex $D^{-}$∙*CD*∙$H_{2}A^{+}$.

The distribution functions $\alpha_{D^{-}}$ and $\alpha_{H_{2}A^{+}}$ describe the relative abundance of the anionic drug form and the diprotonated auxiliary species across the investigated pH range. These functions are calculated from the expressions:

$$\alpha_{D^{-}}=[D^{-}]\left(1+[H^{+}{]}^{2}K_{a1}^{-1}K_{a2}^{-1}+K_{a2}[H^{+}{]}^{-1}\right)$$

$$\alpha_{H_{2}A^{+}}=\left[H_{2}A^{+}\right]\left(1+\beta_{2}^{-1}[H^{+}{]}^{-2}+{\beta_{1}\beta }_{2}^{-1}[H^{+}{]}^{-1}+{\beta_{3}\beta }_{2}^{-1}\left[H^{+}\right]\right)$$

The conditional stability constant of the ternary complex is defined as $\beta^{cond}=\beta_{111}/\left(\alpha_{D^{-}}{\cdot \alpha }_{H_{2}A^{+}}\right)$. This formulation incorporates both drug ionization and protonation equilibria of the auxiliary component. The pH region corresponding to elevated *SC* values therefore reflects conditions where ternary association becomes thermodynamically favorable relative to competing equilibria present in the heterogeneous system.

### Appendix A.7. Generalization of Synergistic Effects in Multicomponent Systems

Similar cooperative effects have been reported in other multicomponent chemical systems involving coupled equilibria and mixed-complex formation [20,30,31,32,33]. These observations indicate that synergistic enhancement originates from collective interactions between simultaneously competing equilibria and not from isolated binary processes. The same thermodynamic principles may therefore contribute to non-additive behavior in both pharmaceutical and non-pharmaceutical heterogeneous systems.

## Appendix B. Solubility Data, Synergistic Coefficients, and Quantitative Consistency Check for the RPG–HPβCD–ARG System

Similar cooperative effects have been reported in other multicomponent chemical systems involving coupled equilibria and mixed-complex formation [20,29,30,31,32,33]. These observations indicate that synergistic enhancement originates from collective interactions between simultaneously competing equilibria rather than from isolated binary processes. The same thermodynamic principles may therefore contribute to non-additive behavior in both pharmaceutical and non-pharmaceutical heterogeneous systems. The saturation solubility data used for estimating the experimental synergistic coefficients are summarized in Table A1, and the corresponding calculated synergistic coefficients are reported in Table A2.

**Table A1:** Saturation solubility of the analyzed complexes in the RPG–HP-β-CD–ARG system, determined in distilled water [8] by three techniques *.

System	Method	Solubility in Water (mg/mL)	Solubility in Water (Recalculated in mol/L)
*RPG*		0.035 ± 0.001	7.73 × 10^−5^
*RPG*—*HP*-*β*-*CD*	PM	0.120 ± 0.001	2.65 × 10^−4^
*RPG*—*HP*-*β*-*CD*—*ARG*	PM	5.6 ± 0.045	1.24 × 10^−2^
*RPG*—*HP*-*β*-*CD*	KM	0.145 ± 0.001	3.20 × 10^−4^
*RPG*—*HP*-*β*-*CD*—*ARG*	KM	8.8 ± 0.078	1.94 × 10^−2^
*RPG*—*HP*-*β*-*CD*	CE	0.220 ± 0.006	4.86 × 10^−4^
*RPG*—*HP*-*β*-*CD*—*ARG*	CE	17.90 ± 0.493	3.96 × 10^−2^

* The ternary complexes were prepared using three techniques: (i) physical mixing of the individual components (PM), (ii) kneading with a binder solution (KM), and (iii) co-evaporation employing a rotary evaporator (CE) [8].

**Table A2:** Calculated synergistic coefficients for the ternary inclusion complexes RPG–HP-β-CD–ARG, derived from the experimental solubility data reported by Vakani et al. (2015) [8].

System	Method	Synergistic Coefficient
*RPG*–*HP*-*β*-*CD*–*ARG*	PM	1.6
	KM	1.7
	CE	1.9

The reported values allow direct experimental estimation of the synergistic coefficient using the definition provided in the main text.

**Table A3:** Quantitative consistency check between experimental [8] and calculated apparent solubilities for the RPG–HPβCD–L-arginine system.

$C_{HP\beta CD}$, mM	$S_{exp}$, mM	$S_{calc}$	Absolute Error, mM	Relative Error, %
0	3.6002	3.6002	≤3.0 × 10^−6^	≤5.0 × 10^−5^
3	4.3625	4.3625	≤3.0 × 10^−6^	≤5.0 × 10^−5^
6	5.1248	5.1248	≤3.0 × 10^−6^	≤5.0 × 10^−5^
9	5.8871	5.8871	≤3.0 × 10^−6^	≤5.0 × 10^−5^
12	6.6494	6.6494	≤3.0 × 10^−6^	≤5.0 × 10^−5^
15	7.4117	7.4117	≤3.0 × 10^−6^	≤5.0 × 10^−5^
